# Fibrin Glue Versus Absorbable Sutures for Conjunctival Closure in Pediatric Strabismus Surgery: A Comparative Study of Clinical Outcomes and AS-OCT Findings

**DOI:** 10.3390/jcm15041531

**Published:** 2026-02-15

**Authors:** Ahmet Yusuf Goktas, Dilara Pirhan

**Affiliations:** Department of Ophthalmology, Faculty of Medicine, Kocaeli University, 41001 Kocaeli, Turkey; drayusufg@gmail.com

**Keywords:** pediatric strabismus surgery, conjunctival closure, fibrin glue, sutures, ocular surface, OSDI-6, AS-OCT, conjunctival thickness

## Abstract

**Background/Objectives:** Conjunctival closure may influence early postoperative comfort and wound healing after pediatric strabismus surgery. We compared fibrin glue with absorbable sutures using anterior segment optical coherence tomography (AS-OCT)-based conjunctival thickness, serial clinical scores, ocular-surface screening, and operative time. **Methods:** We retrospectively reviewed 82 children (5–15 years) who underwent bilateral medial rectus recession. The conjunctiva was closed with 8-0 polyglactin 910 (Vicryl) (suture group, *n* = 40) or fibrin glue (fibrin group, *n* = 42) according to routine practice; right eyes were analyzed. Conjunctival thickness was measured by AS-OCT preoperatively and at week 6. The comfort questionnaire (CQ) score and inflammation score (IS) were recorded on postoperative day 1 and weeks 1, 2, and 6. Total operative time and closure time were obtained from surgical video recordings. Ocular Surface Disease Index-6 (OSDI-6) and non-invasive keratographic break-up time (NIKBUT) were assessed preoperatively and at week 6 in cooperative children (*n* = 62). **Results:** Conjunctival thickness increased in both groups and was slightly higher at week 6 with sutures (*p* < 0.001), with a slightly greater percentage increase (*p* = 0.001). CQ and IS were worse with sutures through week 2 (all *p* < 0.05) and converged by week 6 (both *p* > 0.05). Fibrin glue shortened total operative time (32.75 vs. 35.46 min; *p* < 0.05) and closure time (3.90 vs. 5.35 min; *p* < 0.001). In the ocular-surface subset, OSDI-6 and NIKBUT did not differ between groups at week 6. No infections or granulomas occurred; two early conjunctival wound gaps occurred in the fibrin group and one resolved with topical management, while the other met the dehiscence definition (≥2 mm) and required re-suturing, and both healed without sequelae. **Conclusions:** In pediatric strabismus surgery, fibrin glue demonstrated better early comfort with a modest difference in conjunctival thickness at week 6 along with slightly shorter operative time while clinical scores converged by week 6, and ocular-surface screening outcomes were similar.

## 1. Introduction

Strabismus surgery is one of the most frequently performed ophthalmic procedures in children. Although the primary aim is to restore ocular alignment, perioperative decisions that influence the ocular surface and early postoperative comfort are increasingly recognized as clinically important. Among these, conjunctival closure is a key step, because the conjunctiva, with its rich vascular and immune-cell network and close relationship with Tenon’s capsule, is where postoperative inflammation typically begins and is most pronounced. Conjunctival wound healing is a complex, multistage process in which the method of closure modulates the balance between inflammation and fibrosis. Experimental work has shown a rapid early inflammatory phase, peak fibroblast activity within a few days, and organization of scar tissue within approximately two weeks, with the severity of clinical disease closely related to the degree of conjunctival scarring [1,2,3,4,5,6]. In this setting, conjunctival thickness can be regarded as an objective morphometric parameter reflecting postoperative tissue reaction at the surgical site.

Conjunctival closure has traditionally been performed with absorbable sutures. This approach is familiar and reliable but is often associated with postoperative pain, redness, foreign-body sensation, tearing, and a more prolonged inflammatory course [7,8,9,10]. Multifilament materials, such as polyglactin 910 (Vicryl), may cause a stronger foreign-body reaction, increasing inflammatory cell infiltration and fibroblast activation and potentially leading to pronounced local tissue reaction during the healing period. Over the past two decades, fibrin-based tissue adhesives have emerged as an alternative method of conjunctival closure in strabismus and other anterior segment surgeries. These biological products mimic the physiological coagulation cascade, form a temporary fibrin matrix, and may reduce tissue handling, shorten application time, and lower the inflammatory stimulus at wound edges [9,11,12]. In pediatric patients, baseline ocular surface status may be influenced by factors such as allergic conjunctivitis, blepharitis, meibomian gland dysfunction, and heavy screen use; therefore, ensuring a clinically clean ocular surface is important when interpreting postoperative dry eye-related metrics.

Several clinical studies comparing fibrin glue and sutures for conjunctival closure in strabismus surgery have reported better early comfort and less conjunctival irritation with fibrin, alongside comparable safety [8,9,10,13,14]. However, most available data derive from mixed pediatric–adult cohorts or predominantly adult populations, and outcome assessment has relied largely on subjective symptoms and slit-lamp grading of hyperemia, chemosis, and discharge. Objective, tissue-level evaluation of conjunctival healing, such as quantification of conjunctival thickness with anterior segment optical coherence tomography (AS-OCT), has been incorporated in only a limited number of reports and rarely in a purely pediatric setting [13,14]. Beyond conjunctival closure, recent work has shown that strabismus surgery itself can induce dry eye-like changes in tear break-up time (TBUT), Schirmer scores, meibomian gland morphology, and conjunctival impression cytology findings for up to three months postoperatively, underlining the vulnerability of the pediatric ocular surface to surgical trauma [15]. Likewise, standardized dry eye-related metrics, including the Ocular Surface Disease Index (OSDI) and non-invasive keratographic breakup time (NIKBUT), have rarely been used systematically to characterize the ocular surface response to different conjunctival closure techniques after strabismus surgery.

We aimed to bridge this gap by comparing fibrin glue and Vicryl sutures for conjunctival closure in children undergoing medial rectus recession for horizontal strabismus. Postoperative inflammation and patient comfort were assessed using standardized scoring systems at multiple time points, conjunctival thickness was quantified by AS-OCT, and ocular surface status was evaluated with OSDI and NIKBUT. Total operative time and conjunctival closure time were also recorded. We hypothesized that, compared with sutures, fibrin glue would be associated with less conjunctival thickening on AS-OCT, lower early inflammation and discomfort scores, and shorter operative times, without adversely affecting tear film-related parameters.

## 2. Materials and Methods

### 2.1. Study Design and Ethics

This retrospective, comparative observational study was based on routinely collected clinical and surgical data from pediatric patients operated at the Strabismus Unit, Department of Ophthalmology, Faculty of Medicine, Kocaeli University. At our institution, a standardized postoperative follow-up protocol is strictly implemented for all pediatric strabismus patients. Clinical outcomes, including the comfort questionnaire (CQ) and inflammation scores (IS), are routinely evaluated and documented in the electronic medical records at specified intervals (Day 1, Week 1, Week 2, and Week 6) to monitor recovery quality. Therefore, this study was designed as a retrospective analysis of these prospectively collected routine clinical data.

The study was conducted in accordance with the Declaration of Helsinki and approved by the Kocaeli University Non-Interventional Clinical Research Ethics Committee (2025/459; KU GOKAEK-2025/18/21, approved on 23 September 2025).

### 2.2. Participants and Eligibility

A total of 82 pediatric patients (82 eyes), aged 5–15 years, who underwent bilateral medial rectus recession for horizontal strabismus were included consecutively according to predefined eligibility criteria. All cases were primary surgeries, and none of the children had a history of previous ocular surgery. Patients with ocular pathology other than strabismus (including significant anterior segment disease, corneal opacity, active infection, clinically relevant dry eye, allergic conjunctivitis, blepharitis, or meibomian gland dysfunction) or with relevant systemic disease were excluded. At baseline, none of the included children had clinical signs of allergic conjunctivitis, blepharitis, or meibomian gland dysfunction on slit-lamp examination. All children underwent bilateral surgery with the same conjunctival closure technique in both eyes; however, only the right eye of each patient was included in the analysis to avoid inter-eye correlation.

### 2.3. Study Groups and Conjunctival Closure Techniques

The choice of technique was determined by the availability of the fibrin glue at the time of surgery. Conjunctival closure was performed according to routine clinical practice using one of two techniques. The suture group (Group 1) comprised 40 eyes in which the conjunctival flap was closed with 8-0 polyglactin 910 sutures (Vicryl, Ethicon, Somerville, NJ, USA). The fibrin group (Group 2) comprised 42 eyes in which a commercially available fibrin glue was used (Tisseel VH Fibrin Sealant, Baxter Healthcare Corporation, Deerfield, IL, USA). The closure technique reflected routine practice during the study period. All surgeries were performed by a single experienced strabismus surgeon (D.P.) under general anesthesia using a standardized surgical technique.

### 2.4. Surgical Procedure and Postoperative Regimen

All procedures were performed under sterile operating room conditions. After periocular skin cleansing with 10% povidone-iodine, the eye was draped and an eyelid speculum was inserted. A 6-0 silk traction suture was placed at the limbus to stabilize the globe. A limbal-based conjunctival incision was made nasally to expose the medial rectus muscle. After blunt dissection of the conjunctiva and Tenon’s capsule, the medial rectus was isolated and recessed by the planned amount, and then reattached to its new insertion with standard technique.

For conjunctival closure in the fibrin group, a commercially available fibrin glue was used. After meticulous hemostasis and drying, the two components were applied simultaneously to the bare sclera, and the conjunctiva was re-approximated. The flap was held in apposition to ensure initial adhesion. Since polymerization begins immediately, we allowed at least 2 min for sufficient bonding strength to develop before removing the eyelid speculum. In the suture group, the conjunctiva was closed with interrupted 8-0 polyglactin 910 (Vicryl) sutures.

Postoperatively, all patients received topical moxifloxacin 0.5% and prednisolone acetate 1% eye drops four times daily. Follow-up visits were scheduled on postoperative day 1, week 1, week 2, and week 6, with the same follow-up protocol applied to both groups.

### 2.5. Outcome Measures

Conjunctival thickness (AS-OCT): Conjunctival thickness was measured preoperatively and at the 6-week visit using SPECTRALIS HRA + OCT with the Anterior Segment Module (Heidelberg Engineering, Heidelberg, Germany).

Scans were centered on the horizontal meridian, extending from the limbus to approximately 3 mm posterior, aligned with the muscle insertion. To expose the medial rectus conjunctiva, images were captured with the patient in a temporal gaze position, ensuring head stabilization. Postoperative Week 6 scans were obtained from the same anatomical region for comparison. A line scan protocol was utilized for all acquisitions, and only images with a signal quality of at least 20 dB were included in the evaluation.

**Figure 1:**
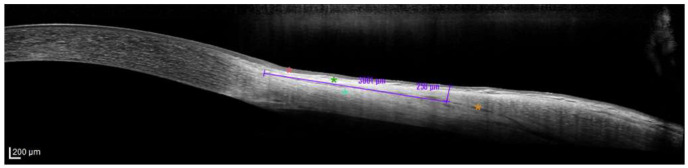
Representative AS-OCT B-scan demonstrating the conjunctival thickness measurement method over the medial rectus muscle. The measurement point was set approximately 3 mm posterior to the limbus (purple horizontal reference line; 3001 µm). Conjunctival thickness was recorded as the perpendicular distance from the conjunctival epithelial surface to the anterior scleral surface at this point (purple vertical line; 250 µm). The red, green, and cyan asterisks indicate the conjunctival epithelium, conjunctival stroma, and sclera, respectively; the orange asterisk marks the anterior scleral surface. (Scale bar: 200 µm.)

Conjunctival thickness measurements were performed on these images, incorporating the conjunctival epithelium, stroma, and Tenon’s capsule, while excluding the episcleral layer. The scleral spur was used as the primary anatomical landmark for the baseline reference. In cases where the scleral spur was not clearly visualized, the echogenic border of the limbus served as the secondary reference point. From this reference point, a linear reference line was drawn along the episclera–conjunctiva interface, extending 3 mm posteriorly along the horizontal axis (Figure 1). Conjunctival thickness was defined as the perpendicular distance from the epithelial surface to the episcleral interface at this 3 mm mark, measured using the device’s caliper tool.

To ensure reliability, three consecutive measurements were taken from the same image for the right eye, and the average value was used for analysis. All measurements were performed by a single investigator who was masked to the conjunctival closure technique. AS-OCT images were de-identified and analyzed in a coded, random order.

Inflammation score and comfort questionnaire: All postoperative assessments were carried out by a single independent ophthalmologist who did not perform the surgeries. This approach was chosen to avoid surgeon bias. Conjunctival inflammation was quantified using a modified grading system adapted from Lee and Kang [10]. A composite Inflammation Score (IS) ranging from 0 to 9 was derived by summing the scores for hyperemia, chemosis, and discharge. Although the visibility of sutures prevented complete blinding during examinations, the examiner strictly followed the standardized grading criteria to maintain consistency.

Postoperative discomfort was evaluated using a Comfort Questionnaire (CQ) adapted from previous pediatric strabismus studies [14,16]. The CQ comprised four domains (pain, redness, stinging/burning, and tearing), each graded on a 0–4 scale (total score 0–16), with higher scores indicating lower comfort. Given the pediatric population, the questionnaire was administered with parental assistance. Parents were instructed to question their children regarding subjective symptoms and to corroborate responses with behavioral observations (e.g., eye rubbing or reluctance to open eyes) to ensure data reliability.

Dry eye-related parameters: Dry eye-related symptoms were screened using the six-item Ocular Surface Disease Index (OSDI-6), as proposed by Pult and Wolffsohn [17]. Each item was scored from 0 to 4, and the total OSDI-6 score was calculated as the unweighted sum of the six items (range: 0–24), with higher scores indicating greater symptom burden. Given the pediatric study population (5–15 years), the questionnaire was administered with parental assistance/supervision, particularly for children younger than 10 years, to ensure accurate comprehension of the items. Given the lack of a fully validated dry eye symptom questionnaire spanning the entire 5–15-year age range, OSDI-6 was used as a pragmatic screening tool and reported descriptively. NIKBUT was measured using the Sirius topography and aberrometry system (CSO, Florence, Italy) under standardized lighting conditions by the same examiner.

Surgical time and complications: All surgeries were recorded digitally as part of the standard protocol. Total surgical time (from eyelid speculum placement to removal) and conjunctival closure time (from initiation to completion of conjunctival closure) were determined by reviewing video recordings with a stopwatch. Two independent observers performed the timing, and the mean of their measurements was used for analysis.

Safety outcomes, including wound infection, conjunctival dehiscence, and granuloma formation, were recorded at each follow-up visit. Small wound gaps <2 mm were recorded and followed conservatively and were not classified as dehiscences.

Conjunctival dehiscence was defined as a visible wound gap ≥2 mm on slit-lamp examination. Management followed a standardized protocol; <2 mm gaps were managed conservatively with topical lubricants, whereas ≥2 mm dehiscence was managed based on clinical relevance, with secondary suturing reserved for clinically significant or persistent defects.

These eyes were retained in the primary comparative analysis according to the initially used closure technique. A sensitivity analysis excluding these two eyes did not materially change the direction or statistical significance of the main outcomes.

### 2.6. Statistical Analysis

The study population included all eligible pediatric patients operated on during the specified review period, consistent with our institutional routine. The sample size therefore reflects the available retrospective cohort during the study period. Only the right eye of each patient was included in the analysis to strictly eliminate statistical bias arising from inter-eye correlation. The allocation to the fibrin glue or suture group was determined by the availability of the fibrin sealant at the time of surgery, rather than surgeon preference or intraoperative findings.

Statistical analyses were performed using IBM SPSS Statistics for Windows, Version 29.0 (IBM Corp., Armonk, NY, USA). Normality was assessed with the Shapiro–Wilk test. Continuous variables are presented as mean ± standard deviation for normally distributed data and as median (25th–75th percentile) for non-normally distributed data; categorical variables are presented as counts and percentages. Between-group comparisons were performed using the independent-samples Student’s *t*-test (parametric) or Mann–Whitney U test (non-parametric), and categorical variables were compared using the chi-square test (or Fisher’s exact test when appropriate). Within-group changes from baseline to week 6 were assessed using the paired-samples *t*-test or Wilcoxon signed-rank test, depending on distribution. Repeated postoperative measures (CQ and IS) across postoperative day 1, week 1, week 2, and week 6 were analyzed using the Friedman test, with post hoc pairwise comparisons adjusted for multiple testing. For post hoc pairwise comparisons across repeated measures, Bonferroni correction was applied. To reduce the risk of overinterpretation driven by multiple comparisons, interpretation focused on the overall longitudinal pattern of CQ/IS rather than isolated nominal *p*-values. OSDI-6 scores were analyzed at the patient level. A *p*-value < 0.05 was considered statistically significant.

## 3. Results

A total of 82 right eyes from 82 children were included (40 in the suture group and 42 in the fibrin group). Baseline demographic characteristics were comparable between groups (Table 1). Conjunctival thickness increased significantly from baseline to postoperative week 6 overall (Table 2). In the intergroup comparison, baseline AS-OCT thickness did not differ, whereas week-6 conjunctival thickness remained higher in the suture group (median 343.5 µm [336.0–355.0] vs. 329.5 µm [319.0–339.0], *p* < 0.001), and the percentage increase from baseline was greater with sutures (43.6% [40.0–52.0] vs. 39.6% [31.0–44.0], *p* = 0.001) (Table 3).

**Figure 2:**
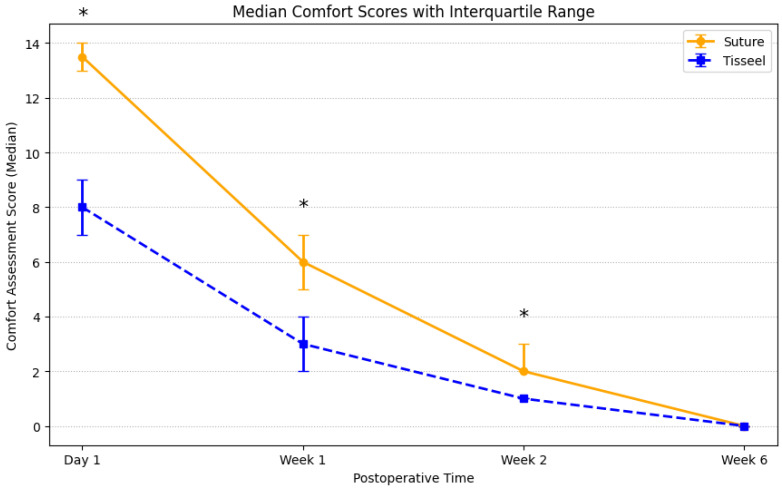
Time course of Comfort Questionnaire (CQ) scores in the suture and fibrin groups at postoperative day 1 and weeks 1, 2, and 6. Data are median (interquartile range); higher scores indicate lower comfort. Error bars represent the 25th–75th percentiles. Asterisks denote statistically significant differences between groups (* *p* < 0.050).

**Figure 3:**
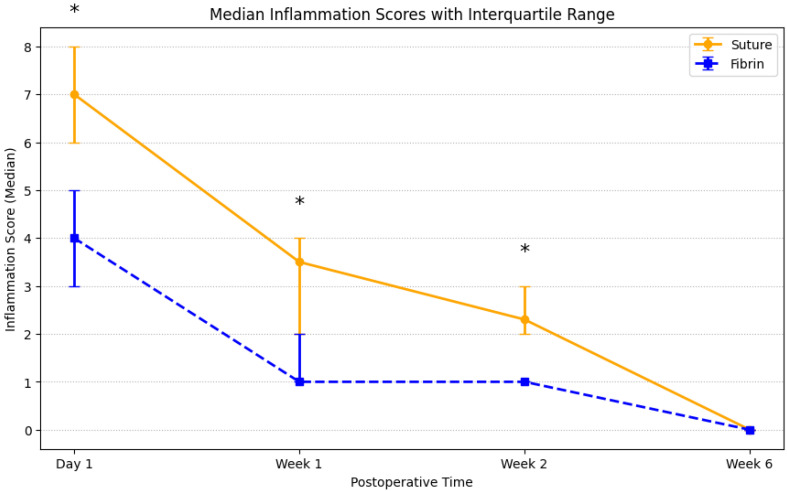
Time course of conjunctival inflammation scores (IS) in the suture and fibrin groups at postoperative day 1 and weeks 1, 2, and 6. Data are median (interquartile range); higher scores indicate more severe inflammation. Error bars represent the 25th–75th percentiles. Higher scores indicate greater postoperative inflammation. Asterisks denote statistically significant differences between groups (* *p* < 0.050).

Postoperative comfort (CQ) and inflammation (IS) improved over time in both groups. Between-group differences were confined to the early postoperative period (day 1 to week 2), favoring fibrin, and the scores converged by week 6 (Table 4 and Table 5; Figure 2 and Figure 3).

Out of the total cohort of 82 children, 62 (76%) were included in the ocular surface analysis because both OSDI-6 and NIKBUT could be obtained preoperatively and at week 6 only in cooperative children with complete measurements. The remaining 20 children were not included because ocular surface testing could not be completed at the week-6 visit due to insufficient cooperation for NIKBUT and/or inability to reliably complete OSDI-6. The rate of missing data due to non-cooperation was statistically similar between the suture and fibrin glue groups (*p* > 0.05). In this analyzed subgroup, neither OSDI-6 nor NIKBUT differed between groups at baseline, and no significant between-group differences were detected at week 6 (Tables S1 and S2). Regarding surgical efficiency, total surgical time was shorter in the fibrin group (32.75 ± 3.95 vs. 35.46 ± 4.94 min; *p* = 0.007), and conjunctival closure time was reduced (3.90 ± 0.69 vs. 5.35 ± 0.96 min; *p* < 0.001) (Table 6). Regarding safety, no wound infection or conjunctival granuloma occurred. Two early conjunctival gaps occurred in the fibrin group; one resolved with topical treatment, and the other met the dehiscence definition (≥2 mm) and required re-suturing under general anesthesia, and both healed without sequelae.

## 4. Discussion

In this retrospective comparative cohort study, we compared Vicryl sutures and fibrin glue for conjunctival closure in pediatric strabismus surgery and also evaluated their impact on conjunctival thickness, inflammation, patient comfort, ocular surface parameters, and operative time. Our main findings were that both techniques led to a significant postoperative increase in conjunctival thickness, but at 6 weeks the conjunctiva remained thicker in the suture group. In contrast, eyes closed with fibrin glue showed a smaller residual increase in conjunctival thickness at week 6. Similarly, patient comfort and inflammation scores were significantly better with fibrin glue during the early postoperative period (day 1 to week 2), with no relevant differences between groups by week 6. Fibrin glue also shortened both conjunctival closure time and total surgical time. No meaningful between-group differences in dry eye-related parameters were detected at the 6-week visit.

The difference in conjunctival thickening between the two techniques is biologically plausible. Vicryl is a braided, multifilament polymer and can trigger a foreign-body response, with macrophage and giant-cell recruitment, longer-lasting inflammation, and ongoing fibroblast activity [18], which may contribute to localized edema and thicker tissue along suture tracks during healing. By contrast, fibrin glue is a biocompatible compound made of fibrinogen and thrombin that mimics physiologic coagulation, provides temporary tissue apposition, and then degrades without leaving long-term foreign material in the wound [19].

Postoperative conjunctival thickening on AS-OCT likely reflects a composite of edema and tissue reaction during healing. In pterygium surgery, graft thickness typically peaks early and then gradually declines over subsequent months [20,21]; however, in our study AS-OCT was obtained only at baseline and at postoperative week 6. At week 6, the between-group difference in conjunctival thickness was statistically significant but small in absolute terms (median difference ~14 µm) and comparable to baseline inter-individual variability (baseline IQR 230–247 µm). Because a minimal clinically important difference for conjunctival thickness in this setting is not established, and because intermediate imaging was not available to characterize early trajectories or potential convergence, this small residual difference should be interpreted cautiously and not as evidence of clinically meaningful fibrosis prevention or structural superiority.

The clinical relevance of these findings pertains primarily to early postoperative recovery. Increased conjunctival thickness together with the mechanical presence of exposed knots may increase eyelid friction during blinking and contribute to lower comfort scores in the early postoperative period [13,14]. This may be particularly relevant in pediatric patients, in whom postoperative conjunctival/Tenon tissue responses can be clinically brisk. Therefore, when reoperation is anticipated in pediatric strabismus, minimizing early tissue reaction may be desirable to help preserve surgical tissue planes, although our study was not designed to evaluate reoperation-related outcomes (e.g., adhesions or scarring). Both groups demonstrated a marked increase in conjunctival thickness from baseline to week 6, indicating that postoperative thickening is a common response after strabismus surgery regardless of closure method. Although the percentage increase was statistically higher in the suture group (43.6% vs. 39.6%), the absolute difference (~4%) is small and should be interpreted cautiously; accordingly, we consider this a modest quantitative difference in tissue response rather than a major clinical advantage.

Our observations align with a recent study by Agarwal et al. [22], who compared markedly shorter conjunctival closure time and lower early objective inflammation with fibrin glue. Using AS-OCT, they also demonstrated a significant increase in conjunctival thickness at 6 weeks in the suture-knot groups compared with baseline, supporting the direction of our objective thickness measurements. Importantly, our study complements data from a homogeneous pediatric cohort, undergoing a standardized procedure performed by one surgeon, using a one eye per-patient approach to avoid inter-eye correlation. In addition, our inclusion of standardized ocular surface metrics (NIKBUT and OSDI-6) broadens the assessment beyond conjunctival appearance to functional surface outcomes in the pediatric setting [22].

Regarding patient comfort, children in the fibrin group experienced substantially less pain, foreign-body sensation, tearing, and irritation in the early postoperative period. This is consistent with previous studies reporting lower pain scores and faster symptom relief with fibrin glue compared with sutures after strabismus or pterygium surgery [8,9,11,13]. In our cohort, fibrin provided a clear comfort advantage during the first two postoperative weeks; however, by week 6, comfort scores were similar between groups, consistent with resolution of early postoperative irritation. CQ and IS differences were confined to the early postoperative period and converged by week 6. Accordingly, the benefit of fibrin glue in our cohort appears limited to early postoperative recovery rather than a sustained between-group difference beyond six weeks.

Inflammation scores (IS) followed a similar course. Hyperemia and edema were less pronounced in the fibrin group at the earliest visits, but this difference had disappeared by week 6, when both groups showed little residual inflammation in. Lee and Kang observed a similar early reduction in inflammation with fibrin during the first three postoperative weeks after strabismus surgery [10]. In our cohort any inflammatory advantage therefore appears confined to the early healing phase rather than being a sustained effect.

Operative time is a clinically relevant consideration in pediatric surgery under general anesthesia. In our study, conjunctival closure with fibrin glue was 1–1.5 min faster than with sutures, reducing total surgical time by approximately 3 min. Although total operative time and conjunctival closure time were shorter with fibrin glue, the absolute differences were small (approximately 2–3 min overall). Accordingly, we interpret this as a modest procedural time difference rather than a major efficiency gain, and its practical relevance may vary depending on the surgical setting and workflow. Despite this time advantage, careful drying, hemostasis, and precise flap apposition remain important when using fibrin glue.

In our cohort, two early conjunctival wound gaps were observed in the fibrin group (2/42 eyes, 4.7%); one met our dehiscence definition (≥2 mm) and required re-suturing, while the other resolved with conservative topical management. No infections or granulomas were observed. Given the low event rate, the present study is not powered to compare rare complications between techniques; therefore, these findings are reported descriptively rather than as evidence of comparative safety. Because wound gap/dehiscence may be influenced by perioperative factors such as moisture control, hemostasis, and flap apposition during glue application, meticulous technique remains essential when using fibrin sealant.

Regarding ocular surface function, we assessed OSDI-6 scores and NIKBUT preoperatively and at 6 weeks and found no meaningful between-group differences at that time point. Previous work suggests that tear film parameters may transiently worsen after ocular surgery and then recover toward baseline within a few months [3]. Conversely, Anand et al. reported greater and more prolonged impairment in tear film break-up time and Schirmer values with sutures compared with fibrin in the early postoperative period [13]. In our study, OSDI-6 was used as a screening tool rather than the full 12-item OSDI, and both OSDI-6 and NIKBUT could only be obtained in a subset of children due to cooperation limitations; moreover, NIKBUT was not assessed at early postoperative visits. These factors reduce sensitivity for detecting subtle or transient differences, and early tear-film instability may have been missed despite the absence of a detectable difference at week 6. Ocular-surface outcomes should be interpreted cautiously. Objective testing (OSDI-6 and NIKBUT) was available only in a cooperative subset (*n* = 62), and at postoperative week 6 we found no detectable between-group difference. Accordingly, we interpret these findings as neutral at week 6, rather than suggesting an ocular-surface protective effect of fibrin glue.

This study contributes to the existing literature in several respects. Most comparative reports on fibrin glue versus sutures in strabismus surgery have involved broad age ranges, combined children and adults, and included a variety of surgical procedures [7,8,9,10,12,13,14]. In our work, by contrast, we focused exclusively on children aged 5–15 years undergoing a single, standardized operation (horizontal strabismus treated with medial rectus recession) performed by one surgeon. Importantly, we combined quantitative AS-OCT measurements of conjunctival thickness with serial comfort and inflammation grading and standardized ocular surface metrics (OSDI, NIKBUT). Currently, pediatric evidence integrating objective AS-OCT conjunctival thickness with serial clinical grading and ocular surface metrics remains limited; our study adds complementary data from a homogeneous pediatric cohort undergoing isolated medial rectus recession, using a standardized surgical approach and a combined structural (AS-OCT) and functional (CQ, IS, OSDI, NIKBUT) outcome framework.

Several methodological considerations contribute to the reliability of our findings. All procedures were performed by a single experienced surgeon using a standard surgical approach, limiting variation owing to operative style. Postoperative assessments and scoring were carried out using a uniform protocol by the same ophthalmologist. All consecutive eligible patients operated during the study period were included.

This study has several limitations. First, because the conjunctival closure technique was chosen according to routine clinical practice rather than protocol-driven randomization, selection bias and unmeasured confounding cannot be excluded, allocation was determined by the availability of fibrin sealant at the time of surgery. Second, AS-OCT imaging was performed only at baseline and at postoperative week 6; therefore, early postoperative thickening trajectories could not be evaluated. In addition, the week-6 between-group difference was small in absolute terms and comparable to baseline inter-individual variability, and a minimal clinically important difference for conjunctival thickness in this setting is not established. Thus, small morphometric differences should be interpreted cautiously and may partly reflect biological variability and measurement variability. Third, ocular surface testing could not be completed in all children due to cooperation limitations, which reduced the effective sample size for OSDI-6 and NIKBUT analyses. Although all children attended their scheduled visits, 20 children could not complete OSDI-6 and/or cooperate for NIKBUT at the week-6 visit and were therefore not included in this sub-analysis. Additionally, because NIKBUT was not measured at early postoperative visits, transient tear-film instability may have been missed. Fourth, although comfort scores and OSDI-6 responses were obtained with parental assistance to maximize accuracy, the reliability of subjective symptom reporting in the younger subset (e.g., 5–7 years) may be lower compared to adolescents. We did not perform a subgroup analysis to validate consistency across different age groups, which may obscure age-related differences in symptom perception. Fifth, postoperative clinical assessments (CQ/IS) were not masked to the closure technique, which may introduce observer bias, although AS-OCT measurements were performed on de-identified images by a masked investigator. Sixth, objective inflammatory biomarkers (e.g., conjunctival impression cytology, goblet cell density, meibography-based indices, or inflammatory mediators) and corneal assessments such as topographic and/or specular microscopy parameters were not included, limiting mechanistic interpretation of ocular surface findings. Finally, this was a single-center study with a short follow-up period, which may restrict generalizability; moreover, the study was not powered to detect between-group differences in rare complications such as wound dehiscence or infection, and the few adverse events observed (including conjunctival wound gaps/dehiscence) should therefore be interpreted cautiously and reported descriptively.

In summary, in pediatric strabismus surgery, fibrin glue may be used as an alternative to Vicryl for conjunctival closure with any benefit largely limited to the early postoperative period. In our cohort, comfort and inflammation scores favored fibrin glue during the first postoperative weeks, and conjunctival closure time (and total operative time) was modestly shorter. By week 6, however, the between-group AS-OCT thickness difference was small, and ocular-surface screening in the cooperative subset did not show a between-group difference; therefore, our data do not support claims of sustained structural or ocular-surface protection. By week 6, however, the between-group AS-OCT thickness difference was small, and ocular-surface screening in the cooperative subset did not show a between-group difference; therefore, our data do not support claims of sustained structural or ocular-surface protection. Vicryl remains a reliable and widely available option, whereas fibrin glue may be preferred when available and when meticulous hemostasis, adequate drying, and precise flap apposition are ensured. Finally, because adverse events were infrequent in this cohort, larger multicenter randomized studies with longer follow-up are needed to evaluate long-term outcomes and to assess rare complications and reoperation-relevant endpoints such as scarring and adhesions.

## Figures and Tables

**Table 1:** Baseline demographic characteristics of the study population.

Parameter	Suture Group (*n* = 40 Patients)	Fibrin Group (*n* = 42 Patients)	Total (*n* = 82 Patients)	*p*-Value
Age, years (mean ± SD)	9.9 ± 3.2	10.2 ± 3.7	10.0 ± 3.4	0.708
Sex, *n* (F/M)	20/20	20/22	40/42	0.879

Abbreviations: SD, standard deviation; F, female; M, male. Values are presented as mean ± SD, median (25th–75th percentile), or *n* (%), as appropriate.

**Table 2:** Change in AS-OCT conjunctival thickness from baseline to postoperative week 6 in all eyes.

Parameter	*n* (Patients)	Mean ± SD	Median (25th–75th Percentile)
AS-OCT conjunctival thickness (µm)			
Preoperative	82	238.5 ± 11.9	239 (230–247)
Postoperative 6 weeks	82	334.5 ± 23.5	336 (324–349)

Data are presented as mean ± standard deviation and median (25th–75th percentile).

**Table 3:** Comparison of AS-OCT conjunctival thickness outcomes between the suture and fibrin groups.

Parameter	Suture Group (*n* = 40 Patients) Median (25th–75th Percentile)	Fibrin Group (*n* = 42 Patients) Median (25th–75th Percentile)	*p*-Value *
Preoperative AS-OCT conjunctival thickness (µm)	240.5 (231.0–250.8)	238.0 (230.0–246.0)	0.318
Postoperative AS-OCT conjunctival thickness, week 6 (µm)	343.5 (336.0–355.0)	329.5 (319.0–339.0)	**<0.001**
Percentage change in AS-OCT conjunctival thickness (%)	43.6 (40.0–52.0)	39.6 (31.0–44.0)	**0.001**

* Data are presented as median (25th–75th percentile). Percent change = (week 6 − baseline)/baseline × 100. AS-OCT = anterior segment optical coherence tomography. Bold values indicate statistically significant *p*-values (*p* < 0.05).

**Table 4:** Postoperative comfort questionnaire (CQ) score over time.

Visit	Suture Group (*n* = 40 Patients) CQ Median (25th–75th Percentile) *	% Change from Day 1 (Suture)	Fibrin Group (*n* = 42 Patients) CQ Median (25th–75th Percentile) *	% Change from Day 1 (Fibrin)	*p*-Value
Day 1	13.5 (13–14)	-	8.0 (7–9)	-	**<0.001**
Week 1	6.0 (5–7)	−55%	3.0 (2–4)	−62%	**<0.001**
Week 2	2.0 (2–3)	−85%	1.0 (1–1)	−88%	**0.040**
Week 6	0 (0–0)	−100%	0 (0–0)	−100%	0.620

* Data are presented as median (25th–75th percentile). Percent change is calculated relative to postoperative day 1 within each group. CQ = Comfort Questionnaire; higher scores indicate lower comfort. *p*-values compare suture vs. fibrin groups at each time point. Bold values indicate statistically significant *p*-values (*p* < 0.05). Negative values indicate a decrease from baseline.

**Table 5:** Postoperative conjunctival inflammation score (IS) over time.

Visit	Suture Group (*n* = 40) Median (25th–75th Percentile)	% Change from Day 1 (Suture)	Fibrin Group (*n* = 42) Median (25th–75th Percentile)	% Change from Day 1 (Fibrin)	*p*-Value
Postoperative day 1	7.0 (6–8)	-	4.0 (3–5)	-	**<0.001**
Week 1	3.5 (2–4)	−50%	1.0 (1–2)	−75%	**<0.001**
Week 2	2.5 (2–3)	−67%	1.0 (1–1)	−75%	**0.030**
Week 6	0 (0–0)	−100%	0 (0–0)	−100%	0.700

Data are presented as median (25th–75th percentile). Higher scores indicate more severe inflammation. Percent changes are calculated relative to postoperative day 1 within each group. *p*-values refer to between-group comparisons at each visit. Negative values indicate a decrease from baseline. Bold values indicate statistically significant *p*-values (*p* < 0.05).

**Table 6:** Surgical outcomes (per patient).

Outcome	Suture Group (*n* = 40)	Fibrin Group (*n* = 42)	*p*-Value
Conjunctival closure time (min), mean ± SD	5.35 ± 0.96	3.90 ± 0.69	**<0.001**
Total operative time (min), mean ± SD	35.46 ± 4.94	32.75 ± 3.95	**0.007**

Values are mean ± SD. Total operative time: speculum in–out; closure time: start–completion of conjunctival closure. Bold values indicate statistically significant *p*-values (*p* < 0.05).

## Data Availability

The data presented in this study are available on reasonable request from the corresponding author. The data are not publicly available due to institutional and ethical restrictions related to patient confidentiality.

## Supplementary Materials

The following supporting information can be downloaded at: https://www.mdpi.com/article/10.3390/jcm15041531/s1. Table S1: OSDI-6 total scores at baseline and week 6 in patients with complete ocular surface data; Table S2: NIKBUT at baseline and week 6 in the same subset.

## Abbreviations

The following abbreviations are used in this manuscript:

AS-OCT	Anterior Segment Optical Coherence Tomography
OSDI	Ocular Surface Disease Index
NIKBUT	Non-Invasive Keratographic Breakup Time
CQ	Comfort Questionnaire
IS	Inflammation Score
MR	Medial Rectus
