# Supplementary material for: Fibrin Glue Versus Absorbable Sutures for Conjunctival Closure in Pediatric Strabismus Surgery: A Comparative Study of Clinical Outcomes and AS-OCT Findings
Source: J Clin Med. 2026 Feb 15;15(4):1531. doi: 10.3390/jcm15041531 (PMC12942599; doi:10.3390/jcm15041531)
Supplement: Supplementary file 1 [file jcm-15-01531-s001.zip › SUPPLEMANTARY FILES_GOKTAS/Suppl. File Table S1_GOKTAS.docx]

**Table S1.** OSDI-6 total scores at baseline and week 6 in the subset with complete ocular surface data (n = 62).

| **Parameter** | **Suture group median (25th-75th)** | **Fibrin group median (25th-75th)** | **p-value*** |
| --- | --- | --- | --- |
| Preoperative OSDI-6 total score (0-24) | 4 (3-4) | 4 (3-4) | 0.427 |
| Postoperative OSDI-6 total score, week 6 (0-24) | 4 (3-4) | 4 (3-4) | 0.321 |
