# Supplementary material for: Fibrin Glue Versus Absorbable Sutures for Conjunctival Closure in Pediatric Strabismus Surgery: A Comparative Study of Clinical Outcomes and AS-OCT Findings
Source: J Clin Med. 2026 Feb 15;15(4):1531. doi: 10.3390/jcm15041531 (PMC12942599; doi:10.3390/jcm15041531)
Supplement: Supplementary file 1 [file jcm-15-01531-s001.zip › SUPPLEMANTARY FILES_GOKTAS/Suppl. File Table S2_GOKTAS.docx]

**Table S2.** NIKBUT at baseline and week 6 in the subset with complete ocular surface data (n = 62).

| **Parameter** | **Suture group median (25th-75th)** | **Fibrin group median (25th-75th)** | **p-value*** |
| --- | --- | --- | --- |
| Preoperative NIKBUT (s) | 12 (11-14) | 12 (11-14) | 0.752 |
| Postoperative NIKBUT, week 6 (s) | 12 (11-14) | 12 (11-14) | 0.937 |
